# Analysis of seasonal variation of antibiotic prescribing for respiratory tract diagnoses in primary care practices

**DOI:** 10.1017/ash.2023.418

**Published:** 2023-09-05

**Authors:** Lacey Serletti, Lauren Dutcher, Kathleen O. Degnan, Julia E. Szymczak, Valerie Cluzet, Michael Z. David, Leigh Cressman, Lindsay W. Glassman, Keith W. Hamilton, for The Centers for Disease Control and Prevention (CDC) Prevention Epicenters Program

**Affiliations:** 1 Department of Medicine, University of Pennsylvania Perelman School of Medicine, Philadelphia, PA, USA; 2 Division of Infectious Diseases, Department of Medicine, University of Pennsylvania Perelman School of Medicine, Philadelphia, PA, USA; 3 Division of Epidemiology, Department of Internal Medicine, University of Utah School of Medicine, Salt Lake City, UT, USA; 4 Division of Infectious Diseases, Nuvance Health, Poughkeepsie, NY, USA; 5 Department of Biostatistics, Epidemiology, and Informatics, University of Pennsylvania Perelman School of Medicine, Philadelphia, PA, USA; 6 Mathematica, Princeton, NJ, USA

## Abstract

**Objective:**

To determine antibiotic prescribing appropriateness for respiratory tract diagnoses (RTD) by season.

**Design:**

Retrospective cohort study.

**Setting:**

Primary care practices in a university health system.

**Patients:**

Patients who were seen at an office visit with diagnostic code for RTD.

**Methods:**

Office visits for the entire cohort were categorized based on ICD-10 codes by the likelihood that an antibiotic was indicated (tier 1: always indicated; tier 2: sometimes indicated; tier 3: rarely indicated). Medical records were reviewed for 1,200 randomly selected office visits to determine appropriateness. Based on this reference standard, metrics and prescriber characteristics associated with inappropriate antibiotic prescribing were determined. Characteristics of antibiotic prescribing were compared between winter and summer months.

**Results:**

A significantly greater proportion of RTD visits had an antibiotic prescribed in winter [20,558/51,090 (40.2%)] compared to summer months [11,728/38,537 (30.4%)][standardized difference (SD) = 0.21]. A significantly greater proportion of winter compared to summer visits was associated with tier 2 RTDs (29.4% vs 23.4%, SD = 0.14), but less tier 3 RTDs (68.4% vs 74.4%, SD = 0.13). A greater proportion of visits in winter compared to summer months had an antibiotic prescribed for tier 2 RTDs (80.2% vs 74.2%, SD = 0.14) and tier 3 RTDs (22.9% vs 16.2%, SD = 0.17). The proportion of inappropriate antibiotic prescribing was higher in winter compared to summer months (72.4% vs 62.0%, *P* < .01).

**Conclusions:**

Increases in antibiotic prescribing for RTD visits from summer to winter were likely driven by shifts in diagnoses as well as increases in prescribing for certain diagnoses. At least some of this increased prescribing was inappropriate.

## Background

Most antibiotics in the United States are prescribed in the outpatient setting.^1^ Respiratory tract diagnoses (RTD) are the most common indication for ambulatory antibiotic prescriptions, up to 50% of which are estimated to be inappropriate.^2,3^

Seasonal fluctuations in prescribing have been demonstrated with wintertime peaks and summertime nadirs for certain diagnoses, including for RTDs.^4–12^ At least some of the increased prescribing for RTDs in the winter months is likely inappropriate.^11^ Because these increases in antibiotic prescribing may drive antibiotic resistance, further understanding of seasonal prescribing patterns is vital to antibiotic stewardship efforts broadly.^5^

Of additional interest is the development of metrics that can serve as proxies for inappropriate antibiotic prescribing in the outpatient setting. For individual prescribers, the proportion of antibiotics prescribed for all RTDs and for the subset of RTDs for which antibiotics are rarely indicated correlates with inappropriate use.^2,11^ On a national level, increased total antibiotic use is associated with greater seasonal fluctuations in antibiotic prescribing.^6–8^ As such, seasonal variation in antibiotic prescribing at the individual prescriber level may also be correlated with inappropriate use.

The primary objective of this study was to determine the appropriateness of antibiotic prescribing by season. Secondary objectives were to describe seasonal variations in prescribing patterns by antibiotic class and diagnosis and to determine whether seasonal variation in rate of prescribing is associated with inappropriate use and with certain prescriber characteristics.

## Methods

### Data collection

A retrospective cohort study was conducted from July 1, 2016, through June 30, 2017, at 32 primary care practices in the University of Pennsylvania Health System, which included 227 primary care providers. These practices serve a diverse population in urban and suburban settings in southeastern Pennsylvania and southern New Jersey with over 90,000 RTD office visits annually. Office visits were eligible for inclusion if: (1) there was an ICD-10 code for at least one RTD; (2) the patient was ≥18 years of age; and (3) the clinician was either an attending physician or advanced practice provider (APP). Only adults were included because only a minority of prescribers saw children. Although teaching practices were included in this study, only office visits in which a patient was seen primarily by an attending physician or APP were included, given that residents were not present in all practices. Office visits were excluded if there was an infection other than an RTD coded in the same encounter. These resulting office visits are subsequently referred to as the *entire cohort*.

Antibiotic(s) prescribed and ICD-10 code(s) for each office visit were collected. Demographics and relevant comorbidities using ICD-10 codes were collected. Charlson comorbidity index for each patient was calculated using methods previously described.^11^ Each visit occurring during a fall or winter month (October through March) was characterized as winter and during a spring or summer month (April through September) was characterized as summer. Prescriber demographics were collected, and ambulatory care practices were characterized as either teaching or nonteaching depending on whether residents saw patients at the clinic and as either urban or nonurban.

Each RTD was further characterized by diagnostic grouping (sinusitis, pneumonia, acute bronchitis, pertussis, otitis media, pharyngitis, and “other RTDs,” which included nonspecific respiratory symptoms such as cough and rhinorrhea and nonspecific diagnoses such as viral upper respiratory tract infection) and by prescribing tier based on the likelihood of that diagnosis to require an antibiotic (tier 1: antibiotic almost always indicated; tier 2: antibiotic sometimes indicated; tier 3: antibiotic rarely indicated) (Supplemental Table 1).^1^

### Determination of Appropriateness of Antibiotic Prescribing

To determine a reference standard of antibiotic appropriateness, encounters were randomly selected from the entire cohort in which there was an oral antibacterial (excluding rifamycins, ethambutol, isoniazid, pyrazinamide, vancomycin, rifaximin, fidaxomicin, fosfomycin, and nitrofurantoin) or intramuscular penicillin prescribed. Sixty prescribers were randomly selected from prescribers with at least 20 RTD visits in the summer months and at least 20 RTD visits in the winter months. Then, 20 office visits for each of these prescribers were randomly selected for a total of 1,200 office visits. Visits were intentionally sampled so that each prescriber had 10 visits in the summer months and 10 visits in the winter months. These office visits are subsequently referred to as the *random cohort.*

To determine the appropriateness of antibiotic prescribing, clinical documentation and diagnostic testing from each office visit in the random subset were reviewed in the electronic health record (EHR) by at least one member of the research team. Diagnostic criteria for determination of appropriateness were adapted from relevant professional society and public health guidelines (Supplemental Table 2).^13–18^

Antibiotics were classified as appropriate if the patient fit the diagnostic criteria for a bacterial respiratory tract infection. Otherwise, they were classified as inappropriate. Only the decision to prescribe an antibiotic was considered when determining appropriateness. Antibiotic choice, dose, and duration were not considered. Chart abstraction was performed using REDCap electronic data capture tools hosted at the University of Pennsylvania.^19^ Fifteen percent of office visits were double-coded by two members of the research team. If there was a discrepancy in the determination of appropriateness, two members of the research team discussed the case and came to a consensus on appropriateness.

### Statistical analysis

For the entire cohort, categorical variables were summarized using frequencies, and continuous variables were summarized using mean and median when appropriate. Proportions of antibiotic prescribing for diagnostic groupings and for tier groupings were compared for the summer and winter months. Similarly, the proportion of prescriptions by antibiotic class was compared for the summer and winter months. The associations between season and antibiotic used and between season and RTD diagnoses were determined using a χ^2^ test.

Each prescriber from the entire cohort with at least 20 RTD visits in summer months and at least 20 RTD visits in winter months was included in an analysis to determine the association of prescriber demographic characteristics with seasonal variation in antibiotic prescribing. To assess whether prescribers with more RTD office visits in winter months or greater changes in RTD visits from summer to winter months were more likely to prescribe antibiotics for these indications in winter months, linear regression was performed on the entire cohort. This analysis evaluated whether the dependent variable prescriber’s seasonal change in percent prescribing from summer to winter is correlated with the prescriber’s total RTD visits in winter months, the absolute seasonal difference in number of RTD visits from summer to winter, and seasonal percent change in RTD visits from summer to winter. The association of prescriber demographic characteristics with the difference in the proportion of RTD visits in which an antibiotic was prescribed between winter and summer months was determined for the entire cohort using *t* test or Wilcoxon rank sum test when appropriate.

The association between season and appropriateness of antibiotic prescribing was determined for the random cohort using a χ^2^ test. To determine if various potential administrative metrics were correlated with inappropriate prescribing, univariate and multivariate fractional logistic regression was used for the random cohort with the proportion of inappropriate prescriptions as the dependent variable and various possible administrative metrics and prescriber characteristic covariates as independent variables. Analysis was performed at the level of the prescriber, and possible administrative metrics included difference in the proportion of prescribing for RTDs between winter and summer months, the proportion of prescribing for all RTDs, and the proportion of prescribing for tier 1, 2, and 3 diagnoses.

Given the large sample size, significance for summary statistics performed on the entire cohort was reported using standardized difference (SD) with SD≥0.1 considered a significant difference. Significance for other analyses was reported using *P*-values with *P* ≤ .05 considered a significant difference except in univariate analyses where more liberal *P* ≤ .20 criteria were used for inclusion in multivariate models.

Data were analyzed using STATA v15.0 (StataCorp, College Station, TX). This study was approved by the Institutional Review Board at the University of Pennsylvania.

## Results

Over the course of the study period, 95,773 office visits were identified, and 6,146 office visits were excluded because another infection other than an RTD was coded. A total of 89,627 unique RTD visits were analyzed as the entire cohort. Demographic characteristics and comorbidities of patients in the entire cohort and random cohort are shown in Table 1. Of all visits in the entire cohort, 38,537 (43.0%) occurred in the summer months and 51,090 (57.0%) occurred in the winter months. Out of all RTD visits, 32,286 (36.0%) were associated with an antibiotic prescription. Of the visits associated with an antibiotic prescription, 11,728 (36.3%) occurred in the summer months and 20,558 (63.7%) occurred in the winter months. There were no significant differences in the proportion of antibiotics prescribed seasonally by class in the entire cohort (Table 2).

**Table 1.** Patient demographics and comorbidities for patients in the entire cohort and the cohort of patients randomly selected for manual review of the appropriateness of antibiotic prescribing for respiratory tract diagnoses compared by standardized difference (SD)

Characteristic	Entire cohort (*N*=32,286)	Random cohort (*N*=1,200)	SD
**Age**, mean (standard deviation)	52 (20)	50 (21)	0.01
**Female gender**, *n* (%)	20,663 (64.0%)	741 (61.8%)	0.02
**Race**			
White, *n* (%)	22,439 (69.5%)	826 (68.8%)	<0.01
Black, *n* (%)	6,662 (20.6%)	241 (20.1%)	<0.01
Asian, *n* (%)	956 (3.0%)	40 (3.3%)	<0.01
Other/unknown, *n* (%)	2,229 (6.9%)	93 (7.8%)	0.01
**Comorbidities**			
Asthma	1,378 (4.3%)	50 (4.2%)	<0.01
Bronchiectasis	67 (0.2%)	2 (0.2%)	<0.01
Cardiomyopathy/congestive heart failure	698 (2.2%)	22 (1.8%)	<0.01
Chronic kidney disease	998 (3.1%)	18 (1.5%)	0.03
Chronic obstructive pulmonary disease (COPD)	625 (1.9%)	30 (2.5%)	<0.01
Cirrhosis	67 (0.2%)	0 (0.0%)	0.08
Coronary artery disease	1,392 (4.3%)	30 (2.5%)	0.02
Diabetes	3,351 (10.4%)	101 (8.4%)	0.01
Human immunodeficiency virus (HIV)	219 (0.7%)	6 (0.5%)	<0.01
Hyperlipidemia	6,275 (19.4%)	192 (16.0%)	
Hypertension	7,750 (24.0%)	262 (21.8%)	0.02
Immunodeficiency (other than HIV, malignancy, and transplant)	159 (0.5%)	0 (0.0%)	0.09
Liver disease (other than cirrhosis)	420 (1.3%)	11 (0.9%)	0.01
Lung disease (other than asthma, bronchiectasis, or COPD)	403 (1.2%)	31 (2.5%)	0.03
Malignancy	2,306 (7.1%)	58 (4.8%)	0.04
Peripheral artery disease	314 (1.0%)	12 (1.0%)	<0.01
Solid organ or bone marrow transplant	116 (0.4%)	6 (0.5%)	<0.01
Stroke or transient ischemic attack	390 (1.2%)	5 (0.4%)	0.01
**Charlson comorbidity index**, median (interquartile range)	0 (0–1)	0 (0–1)	<0.01

**Table 2.** The proportion of antibiotics prescribed by the class during summer and winter months compared using standardized difference (SD)

	Summer antibiotics, *n* (%) (*N*=11,892)	Winter antibiotics, *n* (%) (*N*=20,750)	SD
**Aminopenicillins**	4,264 (35.9%)	7,332 (35.3%)	0.01
**Cephalosporins**	667 (5.6%)	1,111 (5.4%)	0.01
**Macrolides**	4,507 (37.9%)	8,225 (39.6%)	0.03
**Clindamycin**	117 (1.0%)	125 (0.6%)	0.04
**Fluoroquinolones**	1,148 (9.7%)	2,203 (10.6%)	0.01
**Tetracyclines**	879 (7.4%)	1,545 (7.4%)	<0.01
**Trimethoprim-sulfamethoxazole**	256 (2.2%)	373 (1.8%)	0.03
**Metronidazole**	54 (0.5%)	54 (0.3%)	0.02

The frequency of antibiotic prescribing for all RTD visits and by tier and diagnostic groupings for the entire cohort in winter compared to summer months is shown in Table 3. Overall, a greater proportion of RTD visits had an antibiotic prescribed in winter compared to summer months [20,558/51,090 (40.2%) vs 11,728/38,537 (30.4%), SD = 0.21]. A greater proportion of visits were associated with a tier 2 RTD during the winter compared to summer months [15,019/51,090 (29.4%) vs 9013/38,537 (23.4%), SD = 0.14], but less tier 3 RTDs [34,959/51, 090 (68.4%) vs 28,658/38,537 (74.4%), SD = 0.13]. No seasonal difference in the proportion of visits associated with a tier 1 diagnosis was observed. A greater proportion of visits had an antibiotic prescribed in winter relative to summer months for both tier 2 [12,042/15,019 (80.2%) vs 6692/9013 (74.2%), SD = 0.14] and tier 3 diagnoses 8003/34,959 (22.9%) vs 4652/28,658 (16.2%), SD = 0.17]. No seasonal difference in the proportion of antibiotic prescribing for tier 1 diagnoses was observed.

**Table 3.** The proportion of all respiratory tract diagnoses (RTD) by various diagnostic groupings and proportion of office visits in which an antibiotic was prescribed by various diagnostic groupings in summer and winter months including standardized difference (SD)

Diagnostic grouping	Proportion of RTDs made up of various diagnostic groupings			Proportion of office visits in which antibiotic was prescribed by various diagnostic groupings		
	Summer, *n* (%)	Winter, *n* (%)	SD	Summer, *n* (%)	Winter, *n* (%)	SD
**All RTDs**	(NA)	(NA)	NA	11,728 (30.4%)	20,558 (40.2%)	0.21
**Tier**						
Tier 1	866 (2.2%)	1,112 (2.2%)	<0.01	384 (44.3%)	513 (46.1%)	0.04
Tier 2	9,013 (23.4%)	15,019 (29.4%)	0.14	6,692 (74.2%)	12,042 (80.2%)	0.14
Tier 3	28,658 (74.4%)	34,959 (68.4%)	0.13	4,652 (16.2%)	8,003 (22.9%)	0.17
**Diagnosis**						
Bronchitis	2,379 (6.2%)	4,464 (8.7%)	0.11	1,789 (75.2%)	3,404 (76.3%)	0.02
Sinusitis	5,699 (14.8%)	11,018 (21.6%)	0.18	4,852 (85.1%)	9,769 (88.7%)	0.11
Pharyngitis	2,822 (7.3%)	3,593 (7.0%)	0.01	1,520 (53.9%)	1,889 (52.6%)	0.03
Pneumonia	820 (2.1%)	1,061 (2.1%)	<0.01	356 (43.4%)	482 (45.4%)	0.04
Otitis media	1,988 (5.2%)	2,622 (5.1%)	<0.01	1,017 (51.2%)	1,445 (55.1%)	0.08
Pertussis	8 (0.02%)	9 (0.02%)	<0.01	6 (75.0%)	4 (44.4%)	0.06
Other RTDs	28,897 (75.0%)	35,135 (68.8%)	0.14	5,054 (17.5%)	8,694 (24.7%)	0.18

For diagnostic groupings in the entire cohort, a greater proportion of visits with a diagnosis of bronchitis [4,464/51,090 (8.7%) vs 2,379/38,537 (6.2%), SD = 0.11] and sinusitis [11,018/51,090 (21.6%) vs 5,699/38,537 (14.8%), SD = 0.18] occurred in the winter relative to summer months. By contrast, a greater proportion of visits with “other RTDs” was noted in the summer relative to the winter months [28,897/38,537 (75.0%) vs 35,135/51,090 (68.8%), SD = 0.14]. Other diagnoses did not vary seasonally. Greater proportions of antibiotic prescribing were observed in the winter relative to summer months for the diagnoses of sinusitis [9,769/11,018 (88.7%) vs 4,852/5,699 (85.1%), SD = 0.11] and “other RTDs” [8,694/35,135 (24.7%) vs 5,054/28,897 (17.5%), SD = 0.18]. No seasonal difference in the proportions of antibiotic prescribing for other diagnostic groupings was observed.

Overall, 182 prescribers in the entire cohort met the inclusion criteria of having at least 20 RTD visits in the summer months and 20 RTD visits in the winter months. A histogram showing the prescribers’ seasonal change in percent prescribing is shown in Supplemental Figure 1. For a majority of prescribers, there was an increase in percent prescribing for RTDs from summer to winter. In linear regression analyses, seasonal change in percent prescribing on an individual prescriber level was most strongly correlated with absolute increase in RTD visits from summer to winter (*R* ^2^ 0.17, *P* < 0.01), followed by total winter RTD visits (*R* ^2^ 0.15, *P* < .01) and percent change in RTD visits (*R* ^2^ 0.04, *P* < .01).

Associations between prescriber characteristics and seasonal variation in percent prescribing from summer to winter months in the entire cohort are reported in Supplemental Table 3. Family medicine specialty, nonteaching practice, nonurban practice, and board certification ≤15 years were associated with a greater increase in percent prescribing from summer to winter months.

For the random cohort, there was 94% observed agreement and kappa of 0.90 in the determination of the appropriateness of antibiotic prescribing among raters. Overall, the proportion of inappropriate prescribing was 72.4% in the winter months and 62.0% in the summer months (*P* < .01). Certain prescriber characteristics were associated with greater inappropriate prescribing, including family medicine specialty, nonteaching practice, nonurban practice, and APP degree (Supplemental Table 4).

**Figure 1.**
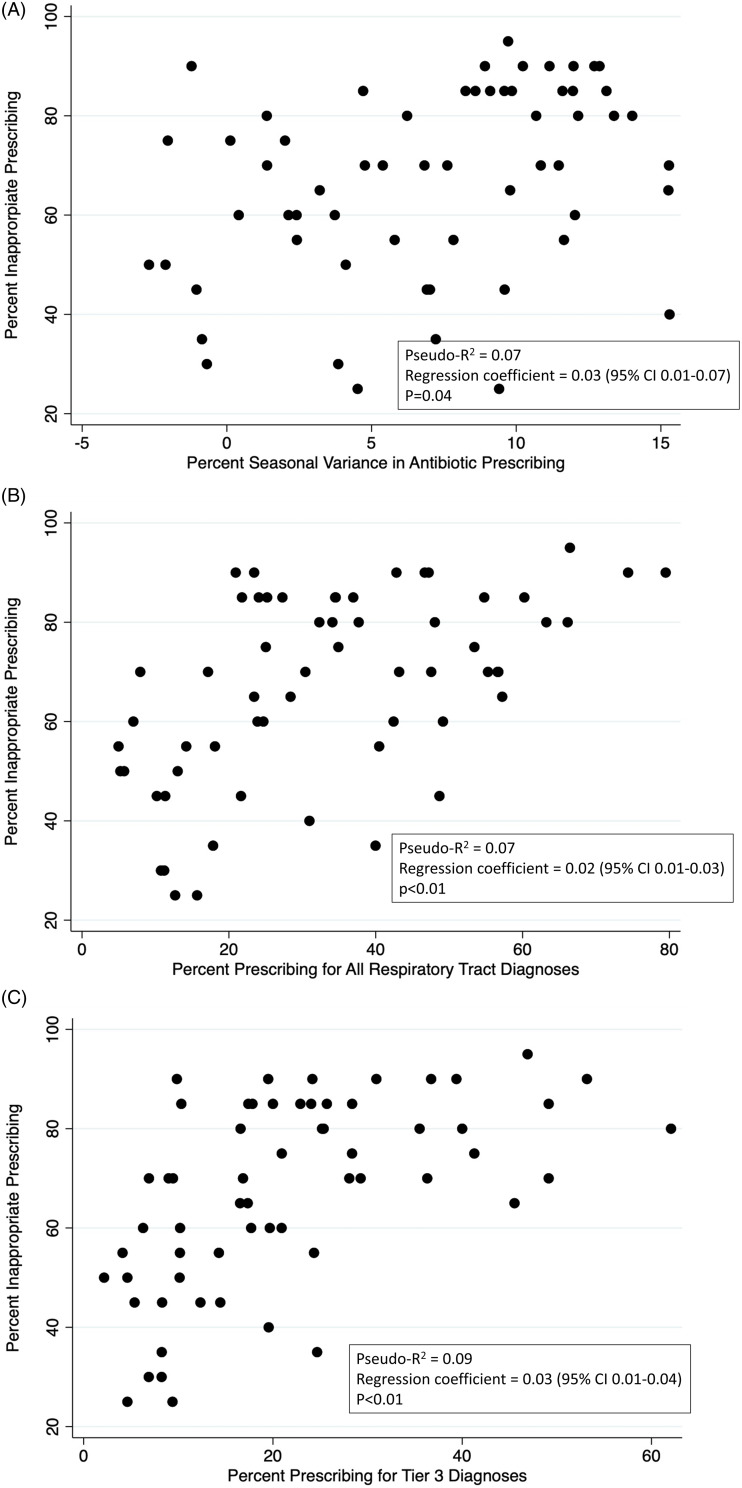
Association of inappropriate antibiotic prescribing for respiratory tract diagnoses (RTDs) with candidate administrative metrics analyzed on a prescriber level for 60 randomly selected prescribers (random cohort): (A) percent seasonal variance in antibiotic prescribing; (B) percent prescribing for all RTDs; and (C) percent prescribing for tier 3 RTDs (results reported from final multivariate model).

Seasonal change in percent prescribing was positively correlated with inappropriate prescribing on a prescriber level even after controlling for prescriber characteristics (pseudo-*R* ^2^ 0.07, *P* < .04) (Supplemental Tables 5 and 6, Figure 1). However, the metrics that were most strongly correlated with inappropriate prescribing were the proportion of prescribing for tier 3 RTDs (pseudo-*R* ^2^ = 0.09, *P* < .01) and for all respiratory visits (pseudo-*R* ^2^ = 0.07, *P* < .01).

## Discussion

We observed greater proportions of antibiotic prescribing in the winter months relative to the summer months. We also observed greater proportions of inappropriate prescribing in the winter months, suggesting that at least part of the wintertime increase is driven by inappropriate prescribing. In particular, we observed greater proportions of antibiotic prescribing for tier 2 and tier 3 diagnoses, specifically for sinusitis, otitis media, and “other RTDs”, which included nonspecific respiratory symptoms such as cough and rhinorrhea and nonspecific diagnoses such as viral upper respiratory tract infection.

Multivariate analysis indicated that potential prescriber-level administrative metrics are correlated with inappropriate prescribing, including (listed in order of decreasing correlation): percent prescribing for tier 3 RTDs, percent prescribing for all RTDs, and seasonal prescribing variance for RTDs. However, the pseudo-*R* ^2^ value for all of these potential administrative metrics were all <0.2, indicating that, although significantly correlated with inappropriate prescribing, they are imperfect correlates. Nevertheless, they still may be useful indicators of inappropriate antibiotic prescribing for RTDs.

We observed no significant changes in prescribing for antibiotics by class from summer to winter months. Though seasonal fluctuations in antibiotic prescribing have previously been shown to be driven by increased prescribing of aminopenicillins and macrolides in the winter months, the observed wintertime increase likely reflects the contribution of seasonal prescribing for RTDs.^4,5,20^ By focusing our analysis on RTDs alone, we likely mitigated this effect and consequently saw no substantial shifts in antibiotic classes.

One possible explanation for the increase in inappropriate prescribing in winter months is decision fatigue, the phenomenon where a person’s ability to make decisions declines in quality after a long period of decision making. As visits for RTDs increase in winter months and clinicians must make more decisions about prescribing antibiotics, the quality of these decisions may deteriorate over time.^21^ Decision fatigue may be exacerbated by emotional exhaustion arising from countering patient demand for unnecessary antibiotics.^22,23^ A similar phenomenon has been observed during individual clinic sessions with clinicians prescribing antibiotics more frequently at the end of a clinic session relative to the beginning.^24^ In this study, there was a significant association between a clinician’s inappropriate antibiotic prescribing and the difference in their total RTD visits from summer to winter, their percent increase in total RTD visits from summer to winter, and their total number of RTD visits in the winter, suggesting the possibility of decision fatigue. However, additional investigation is needed to understand seasonal variation in clinical decision-making about antibiotics.

Another possible contributing factor is recency bias, a cognitive bias that causes decisions to be disproportionately impacted by the most recent events.^25–27^ It is clear that clinicians see a greater number of patients with RTDs in the winter months, and it is also possible that they see more infections where antibiotics are warranted as well. More visits with bacterial infections may, in turn, drive inappropriate prescribing through recency bias. In this study, there was a decrease in tier 3 diagnoses and an increase in tier 2 diagnoses from summer to winter months. These changes could reflect accurate differences in patient presentations, but clinicians may also be primed to expect a diagnosis that may require antibiotics in the winter months and so are more likely to assign a tier 2 RTD instead of a tier 3 RTD. Alternatively, having decided to prescribe an antibiotic a priori, they may adjust their coding to justify the prescription.

Finally, it is possible that individual clinician well-being may play a role in the increased antibiotic prescribing observed in the winter months. A retrospective cohort study found that clinicians’ self-reported level of anxiety and depression was positively associated with proportions of inappropriate prescribing for acute respiratory tract infections.^28^ Anxiety and depression may be expected to peak in the winter months due to the contribution of seasonal affective disorder.^29^

In addition to prescriber factors, patient factors may also contribute to increased proportions of antibiotic prescribing in winter months. It is possible that patients’ expression of the number and/or severity of symptoms may influence clinicians’ perceptions of the likelihood of bacterial infection. Similarly, patients may be more likely to specifically appeal for antibiotics in the winter months.^22^ These potential differences in how symptoms are communicated by the patient and perceived by the prescriber could also drive differences in coding more or less specific diagnoses. Additional studies are necessary to investigate the language around patients’ descriptions of symptoms, suggested diagnoses, or requests for antibiotics.

It was previously observed that certain prescriber characteristics were associated with greater inappropriate prescribing, specifically APP degree, family medicine specialty, fewer years in practice, nonteaching practice, and nonurban practice.^11^ Many of these characteristics were also associated with greater inappropriate prescribing in this study that intentionally sampled visits in summer and winter months, specifically family medicine specialty, APP degree, nonteaching practice, and nonurban practice. Additional investigation is needed to determine how prescriber characteristics impact the appropriateness of antibiotic prescribing and how antibiotic stewardship interventions targeted at these specific groups impact antibiotic prescribing.

This study has several limitations. First, it was a retrospective single health-system study, possibly limiting the generalizability of results. Second, RTD metrics and analyses were based on diagnostic codes, which may not be accurate representations of actual diagnoses. Although the determination of appropriateness was based on manual chart review, many of the analyses still depended on diagnostic codes. Third, in determining the association of appropriate prescribing with various characteristics, the small number of prescribers in the random subset precluded multivariate analysis and investigation of the association of different characteristics with each other. Fourth, this study included only adult patients so results should not be applied to pediatric patient populations.

In conclusion, there was a greater proportion of tier 2 compared to tier 3 RTDs in winter months; there were greater proportions of inappropriate prescribing overall in the winter months; and variation in prescribing from summer to winter months was positively correlated with inappropriate prescribing at the level of the prescriber. More investigation is needed to determine the factors influencing seasonal variations in diagnosis coding and in antibiotic prescribing patterns. A better understanding of seasonal variation in antibiotic prescribing for RTDs may facilitate antibiotic stewardship interventions to reduce inappropriate prescribing in these settings.

## Supporting information

Serletti et al. supplementary material Serletti et al. supplementary material

## Supplementary material

For supplementary material accompanying this paper visit http://doi.org/10.1017/ash.2023.418. click here to view supplementary material
