# Supplementary material for: Analysis of seasonal variation of antibiotic prescribing for respiratory tract diagnoses in primary care practices
Source: Antimicrob Steward Healthc Epidemiol. 2023 Sep 5;3(1):e147. doi: 10.1017/ash.2023.418 (PMC10523546; doi:10.1017/ash.2023.418)
Supplement: Serletti et al. supplementary material [file S2732494X23004187sup001.docx]

**Supplemental Table 1.** ICD-10-CM codes for respiratory tract diagnoses.^1^

| **Tier** | **ICD-10 Diagnostic Codes** |
| --- | --- |
| Tier 1 (antibiotics almost always indicated) | A20.2 (pneumonic plague); A21.2 (pulmonary tularemia); A22.1 (pulmonary anthrax); A36.0, A36.1 (diphtheria); A37 (whooping cough); A48.1 (Legionnaire’s disease); A70 (*Chlamydia psittaci* pneumonia); D57.01, D57.211, D57.411, D57.811 (acute chest syndrome); H70 (mastoiditis); J02.0, J03.0 (streptococcal pharyngitis/tonsillitis); J13-J18 (bacterial pneumonia); J36 (peritonsillar abscess); J39.0 (retropharyngeal/parapharyngeal abscess); J39.1 (abscess of pharynx); J85 (lung abscess); J86 (pyothorax); J95.02 (tracheostomy infection); J95.851 (ventilator-associated pneumonia); J98.51 (mediastinitis) |
| Tier 2 (antibiotics sometimes indicated) | A38 (scarlet fever); H66, H67 (otitis media); J01 (acute sinusitis); J02.8, J02.9 (acute pharyngitis); J03.8, J03.9 (acute tonsillitis); J31.2 (chronic tonsillitis); J32 (chronic sinusitis); J44.0, J44.1 (chronic obstructive pulmonary disease with acute exacerbation) J47.0, J47.1 (bronchiectasis with acute exacerbation); O29.01, O74.0, O89.0 (aspiration pneumonitis) |
| Tier 3 (antibiotics rarely indicated) | B01.2, B05.2, B25.0, J12 (viral pneumonia/pneumonitis); B34.0, B34.2, B97.0, B97.21, B97.29, B97.4, B97.81, B97.89 (viral infection); B44.8 (aspergillosis); D86.0, D86.2 (sarcoidosis), H65 (serous/chronic otitis media); H68, H69 (Eustachian tube disorder); J00 (acute nasopharyngitis), J04 (acute laryngotracheitis), J05 (acute obstructive laryngitis); J06 (acute upper respiratory infection); J09-J11 (influenza); J20-J22 (acute bronchitis/bronchiolitis); J30, J31.0 (rhinitis); J31.1 (chronic nasopharyngitis); J33, J34 (disorders of nose and sinuses), J35 (disorders of tonsils); J37, J38, J39.2-J39.9 (disorders of larynx/pharynx/trachea); J40 (bronchitis unspecified); J41, J42 (chronic bronchitis); J43, J44.9 (chronic obstructive pulmonary disease); J45 (asthma); J47.9 (bronchiectasis without exacerbation), J60-J66 (pneumoconiosis); J67-J70 (pneumonitis); J80 (acute respiratory distress syndrome); J81 (pulmonary edema); J82 (pulmonary eosinophilia); J84 (pulmonary fibrosis); J90-J94 (disorders of pleura); J95 except J95.02 and J95.811 (respiratory complications following surgery); J96 (acute respiratory failure); J98 except J98.51, J99 (other respiratory disorders); M34.81 (nasal mucositis); R04.2, R04.8, R04.9 (bleeding in respiratory tract); R05 (cough); R06 (abnormalities of breathing); R07.0 (throat pain); R09.0 (hypoxia); R09.1 (pleurisy); R09.3 (abnormal sputum); R09.81 (nasal congestion); R09.82 (post-nasal drip); T78.4 (allergy) |

1. Adapted from: Fleming-Dutra KE, Hersh AL, Shapiro DJ, et al. Prevalence of inappropriate antibiotic prescriptions among US ambulatory care visits, 2010-2011. JAMA. 2016;315(17):1864-1873.

**Supplemental Table 2**. Criteria used to determine appropriateness of antibiotic prescribing in random cohort by manual review of electronic health record.

| **Diagnosis** | **Criteria** |
| --- | --- |
| Pneumonia^1^ | - At least one of the following: fever; cough; pleuritic pain **AND** - Radiographic evidence of pneumonia **OR** compelling reason(s) to make clinical diagnosis of pneumonia and avoid chest radiography |
| Sinusitis^2^ | - One of the following: purulent nasal/sinus drainage; facial/sinus pain **AND** - One of the following: persistent symptoms or signs lasting ≥10 days without improvement; severe symptoms such as fever >39°C (102°F) that last for ≥3 days; symptom/sign that could indicate complication such as preseptal/orbital cellulitis; double worsening (symptoms initially improved but then became worse) |
| Pharyngitis^3^ | - Sore throat **AND** - Positive rapid test or culture for group A *Streptococcus* **OR** if testing not able to be performed at least 3 of the following: tonsillar exudates, tender anterior cervical lymphadenopathy, fever, absence of cough |
| Otitis media^4^ | - Ear pain **AND** - At least one of the following: redness/erythema of tympanic membrane; bulging of tympanic membrane; symptoms/signs of severe infection (fever, hearing loss, tenderness of mastoid bone, or purulence of ear canal) |
| Pertussis^5^ | - At least one of the following: cough ≥2 weeks with at least one qualifying symptom (paroxysms of coughing, post-tussive vomiting, inspiratory whoop, apnea); positive testing for *Bordetella pertussis* (culture, polymerase chain reaction, serology) |
| Bronchitis^6^ | - At least one of the following: chronic obstructive pulmonary disease; bronchiectasis **AND** - Flare of underlying condition **AND** - Increased cough with purulent sputum |

1. Adapted from: Mandell LA, Wunderink RG, Anzueto A, et al. Infectious Diseases Society of America/American Thoracic Society consensus guidelines on the management of community-acquired pneumonia in adults. *Clin Infect Dis* 2007;44 Suppl 2:S27-72.
2. Adapted from: Chow AW, Benninger MS, Brook I, et al. IDSA clinical practice guideline for acute bacterial rhinosinusitis in children and adults. *Clin Infect Dis* 2012;54:e72-e112.
3. Adapted from: Shulman ST, Bisno AL, Clegg HW, et al. Clinical practice guideline for the diagnosis and management of group A streptococcal pharyngitis: 2012 update by the Infectious Diseases Society of America. *Clin Infect Dis* 2012;55:e86-102.
4. Adapted from: Lieberthal AS, Carroll AE, Chonmaitree T, et al. The diagnosis and management of acute otitis media. *Pediatrics* 2013;131:e964-999.
5. Adapted from: Pertussis (Whooping Cough). Centers for Disease Control and Prevention webside. <https://www.cdc.gov/pertussis/surv-reporting.html>. Published 2022. Accessed September 24, 2020.
6. Adapted from: Global Initiative for Chronic Obstructive Lung Disease. Global strategy for the diagnosis, management, and prevention of chronic obstructive pulmonary disease 2018 report. https://goldcopd.org/wp-content/uploads/2017/11/GOLD-2018-v6.0-FINAL-revised-20-Nov_WMS.pdf. Published 2018. Accessed June 7, 2020.

**Supplemental Figure 1.** Seasonal change between summer and winter months in overall percent prescribing for 182 included prescribers.


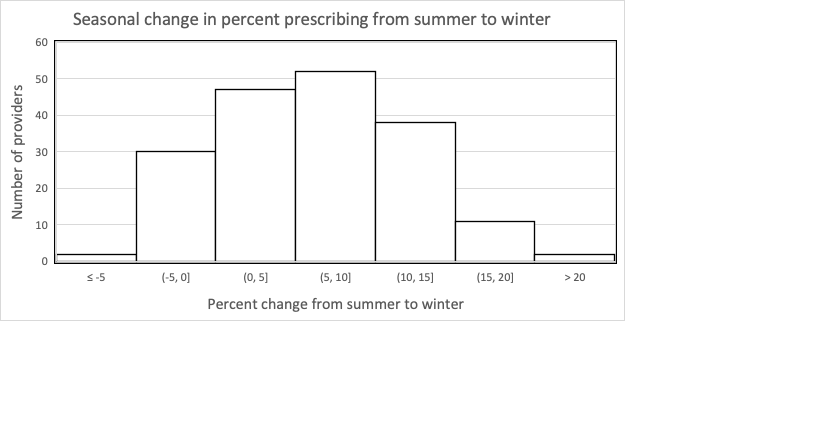


**Supplemental Table 3.** Association of seasonal percent antibiotic prescribing for respiratory tract diagnoses with prescriber characteristics from entire cohort.^1^

| **Prescriber characteristic** | **Mean % prescribing in summer months (standard deviation)** | **p-value** | **Mean % prescribing in winter months (standard deviation)** | **p-value** | **Mean absolute increase in % prescribing from summer to winter (standard deviation)** | **p-value** |
| --- | --- | --- | --- | --- | --- | --- |
| **Degree**  Physician  APP | 20.9% (15.8)  36.9% (19.8) | <0.01 | 27.4% (19.1)  42.8% (21.4) | <0.01 | 6.5% (5.9)  5.8% (6.1) | 0.50 |
| **Gender**  Male  Female | 20.7% (17.5)  27.3% (18.1) | 0.02 | 27.1% (20.4)  33.7% (20.4) | 0.03 | 6.4% (6.3)  6.3% (5.8) | 0.92 |
| **Specialty**  IM  FM | 17.5% (13.2)  34.6% (19.3) | <0.01 | 22.5% (15.4)  42.8% (21.2) | <0.01 | 5.1% (5.7)  8.2% (5.8) | <0.01 |
| **Board Certification**  ≤15 years  >15 years | 27.2% (19.1)  21.1% (16.4) | 0.03 | 33.3% (21.2)  28.1% (20.3) | 0.10 | 6.0% (5.8)  6.8% (6.2) | 0.03 |
| **Teaching status**  Teaching  Nonteaching | 10.7% (6.8)  31.8% (17.9) | <0.01 | 13.6% (8.7)  39.9% (19.4) | <0.01 | 2.9% (4.8)  8.1% (5.7) | <0.01 |
| **Practice setting**  Urban  Nonurban | 14.6% (11.1)  35.8% (17.8) | <0.01 | 18.5% (13.1)  44.9% (18.4) | <0.01 | 3.9% (5.0)  9.1% (5.7) | <0.01 |

1. Abbreviations: APP = advanced practice provider; IM = internal medicine; FM = family medicine

**Supplemental Table 4**. Prescriber demographic characteristics and association with inappropriate antibiotic prescribing for 60 randomly selected providers (random cohort) compared by standardized difference (SD).

| **Prescriber characteristic** | **N (%)** | **Median % inappropriate (interquartile range)** | **p-value** |
| --- | --- | --- | --- |
| **Prescriber degree** | | | |
| Physician | 45 (75%) | 65% (55-73) | <0.01 |
| APP | 15 (25%) | 70% (55-85) |  |
| **Prescriber gender** | | | |
| Male | 22 (37%) | 68% (51-80) | 0.06 |
| Female | 38 (63%) | 78% (61-85) |  |
| **Prescriber specialty** | | | |
| Internal medicine | 38 (63%) | 65% (50-80) | 0.02 |
| Family medicine | 22 (37%) | 80% (66-85) |  |
| **Board certification** | | | |
| >15 years | 28 (47%) | 73% (55-85) | 0.10 |
| ≤15 years | 32 (53%) | 65% (53-80) |  |
| **Teaching status** | | | |
| Teaching practice | 12 (20%) | 45% (30-50) | <0.01 |
| Nonteaching practice | 48 (80%) | 75% (65-85) |  |
| **Practice setting** | | | |
| Urban practice | 26 (43%) | 55% (45-70) | <0.01 |
| Nonurban practice | 34 (57%) | 80% (70-85) |  |

**Supplemental Table 5.** Univariable analysis of association of potential administrative respiratory tract diagnosis (RTD) metrics with inappropriate antibiotic prescribing based on manual medical record review for 60 randomly selected prescribers.^1,2^

| **Independent Variables** | **Regression coefficient** | **95% CI** | ***p-*value** | **Pseudo R^2^** |
| --- | --- | --- | --- | --- |
| **Candidate Metrics** | | | | |
| Percent Prescribing for tier 1 RTDs | -0.40 | -1.14-0.37 | 0.32 | 0.002 |
| Percent Prescribing for tier 2 RTDs | 1.40 | 0.55-2.26 | <0.01 | 0.02 |
| Percent Prescribing for tier 3 RTDs | 0.04 | 0.03-0.05 | <0.01 | 0.04 |
| Percent Prescribing for all RTDs | 0.03 | 0.02-0.03 | <0.01 | 0.04 |
| Seasonal prescribing variance^3^ | 0.06 | 0.02-0.10 | <0.01 | 0.02 |
| **Candidate Covariates** | | | | |
| Median Charlson comorbidity index for prescriber’s RTD patients | -0.63 | -1.04- -0.21 | 0.07 | 0.02 |
| Teaching status (teaching v. non-teaching) | -1.35 | -1.68- -1.02 | <0.01 | 0.05 |
| Practice location (urban v. non-urban) | -0.95 | -1.33- -0.56 | <0.01 | 0.04 |
| Degree (physician v. APP) | -0.33 | -0.80-0.14 | 0.06 | 0.01 |
| Specialty (IM v. FM) | -0.42 | -0.86-0.03 | 0.07 | 0.01 |
| Gender (female v. male) | 0.47 | 0.04-0.90 | 0.03 | 0.01 |
| Board certification year (≤15 years v. >15 years) | 0.27 | -0.17-0.72 | 0.22 | 0.01 |

1. Univariable fractional logistic regression used to determine association of several metrics with the dependent variable inappropriate antibiotic prescribing for RTDs as determined by manual medical record review.
2. Abbreviations: APP = advanced practice provider; IM = internal medicine; FM = family medicine
3. Difference between percent antibiotic prescribing for RTDs in winter and percent antibiotic prescribing for RTDs in summer

**Supplemental Table 6.** Final multivariable models of association of potential administrative respiratory tract diagnosis (RTD) metrics with inappropriate antibiotic prescribing based on manual medical record review for 60 randomly selected prescribers.^1,2^

| **Candidate metric listed with prescriber characteristic covariates from final multivariable model^1,2^** | **Regression coefficient** | **95% CI** | ***p-*value** | **Pseudo R^2^** |
| --- | --- | --- | --- | --- |
| Percent Prescribing for tier 2 RTDs  Teaching status (teaching v. non-teaching)  Gender (female v. male) | 0.80  -1.12  0.60 | -0.07-1.67  -1.50- -0.73  0.26-0.95 | 0.07  <0.01  <0.01 | 0.06 |
| Percent Prescribing for tier 3 RTDs  Teaching status (teaching v. non-teaching)  Gender (female v. male) | 0.03  -1.01  0.49 | 0.01-0.04  -1.43- -0.51  0.24-0.75 | <0.01  <0.01  <0.01 | 0.09 |
| Percent Prescribing for all RTDs  Teaching status (teaching v. non-teaching)  Prescriber gender (female v. male) | 0.02  -0.89  0.62 | 0.01-0.03  -1.28- -0.49  0.31-0.92 | <0.01  <0.01  <0.01 | 0.07 |
| Seasonal prescribing variance  Teaching status (teaching v. non-teaching)  Prescriber gender (female v. male) | 0.03  -1.21  0.51 | 0.01-0.07  -1.57- -0.85  0.20-0.83 | 0.04  <0.01  <0.01 | 0.07 |

1. Multivariable fractional logistic regression was used to determine association of several metrics with the dependent variable inappropriate antibiotic prescribing for RTDs as determined by manual medical record review.
2. Candidate covariate prescriber board certification year was not included in final multivariable models because p>0.20 in univariable analysis; candidate covariates prescriber degree, prescriber specialty, and median Charlson comorbidity index were not included in final multivariable models because they were not statistically significant when included in multivariable models; and candidate covariate practice location was not included in final multivariable models because it was co-linear with teaching status.
3. Difference between percent antibiotic prescribing for RTDs in winter and percent antibiotic prescribing for RTDs in summer.
